# Self-Perception of Physical Problems in Patients with Non-Vascular Type of Ehlers–Danlos Syndrome: A Qualitative Study

**DOI:** 10.3390/healthcare12232392

**Published:** 2024-11-28

**Authors:** Inmaculada C. Palomo-Toucedo, María Reina-Bueno, Pedro V. Munuera-Martínez, María del Carmen Vázquez-Bautista, Gabriel Domínguez-Maldonado, Fatima Leon-Larios

**Affiliations:** 1 Department of Podiatry, Faculty of Nursing, Physiotherapy and Podiatry, Universidad de Sevilla, 41009 Seville, Spain; ipalomo@us.es (I.C.P.-T.); mreina1@us.es (M.R.-B.); carmenvaz@us.es (M.d.C.V.-B.); gdominguez@us.es (G.D.-M.); 2 Department of Nursing, Faculty of Nursing, Physiotherapy and Podiatry, Universidad de Sevilla, 41009 Seville, Spain; fatimaleon@us.es

**Keywords:** Ehlers–Danlos syndrome, hypermobility, fatigue, chronic pain

## Abstract

Background/Objectives: Ehlers–Danlos syndrome is a group of inherited connective tissue disorders characterized by joint hypermobility, skin hyperextensibility, and tissue fragility. Ehlers–Danlos syndrome is associated with a broad spectrum of clinical manifestations, including chronic pain, severe fatigue, and a range of physical and psychological complications. This study aims to identify, in patients with non-vascular type of Ehlers–Danlos syndrome, the most common physical symptoms, the impact of these symptoms on daily life, and individuals’ perceptions of their health. Methods: A qualitative descriptive study based on content analysis was employed, reviewing 24 individual interviews to gain a comprehensive understanding of participants’ experiences. The study was conducted in accordance with the COREQ (Consolidated Criteria for Reporting Qualitative Research) guidelines, which include a 32-item checklist commonly used in qualitative research. Results: Four main themes were identified: (1) Common physical symptoms, (2) Impact on daily life, (3) Impact on social and family relationships, and (4) Health perception and well-being. Conclusions: The analysis of the interviews reveals that individuals with Ehlers–Danlos syndrome face significant physical and emotional challenges. Physical symptoms, particularly chronic pain, fatigue, and joint issues, severely impact their ability to lead a normal life. These symptoms, along with perceived uncertainty and stress, contribute to a reduced quality of life, affecting both physical and emotional well-being.

## 1. Introduction

Ehlers–Danlos Syndrome (EDS) is a connective tissue disorder caused by genetic mutation affecting some collagen protein synthesis [1]. Among its various subtypes, hypermobile EDS (hEDS) is the most common, accounting for 80% to 90% of diagnosed cases, characterized by joint hypermobility, skin hyperextensibility, and tissue fragility. Since connective tissue is found in a multitude of tissue structures, disorders of the musculoskeletal system such as joint dysautonomia and subluxations; dermatological, digestive, gynecological, cardiovascular disorders; and emotional disorders, including anxiety and depression, are recorded [2,3]. Teran-Wodzinski and Kurman [4] also included gait disturbances, balance problems, and reduced joint proprioception. This plethora of disturbances and symptomatology is a common referenced reason for diagnostic delay, as Tapasak’s autobiographical article showed [5].

It is also associated with a wide range of clinical manifestations, including chronic pain, recurrent joint dislocations, extreme fatigue, and various non-musculoskeletal complications such as cardiovascular, digestive, and psychological issues [6,7]. Classic and hEDS subtypes present similar clinical manifestations [4].

Vascular EDS is associated with other characteristic and potentially life-threatening complications such as spontaneous arterial dissections, intestinal rupture, and uterine rupture [8].

Chronic pain is one of the most prevalent and debilitating symptoms in patients with EDS, significantly affecting their quality of life and daily functioning. Studies have shown that pain in EDS tends to be widespread, affecting multiple regions of the body and is often related to hypermobility and recurrent joint dislocations [1,7]. Additionally, the severity of pain is correlated with poor sleep quality, which worsens fatigue and limits the patients’ ability to perform daily activities [6]. Voermans et al. found that severe pain is closely linked to hypermobility and previous surgeries, highlighting the need for pain management that is more focused on the specific factors of EDS [1]. The fatigue, reported by the majority of patients with EDS, is persistent and severe, affecting their ability to maintain an active life and participate in social activities [7,9].

The identification of common physical symptoms in patients with EDS is essential, as these symptoms impact not only daily functionality but also influence the overall perception of health and well-being of the patients. A detailed understanding of these symptoms can improve early diagnosis and the development of personalized management strategies that address the specific needs of each patient [10,11]. In the study of Rocchetti et al. [12], is highlighted how physical symptoms, such as digestive issues and sleep dysfunction, profoundly affect the quality of life of patients with EDS, emphasizing the need for proper diagnosis and a comprehensive therapeutic approach.

In addition to physical symptoms, EDS has a significant impact on mental health and psychological well-being. Various studies have documented a high prevalence of anxiety and depression in patients with EDS, especially in those with hEDS, which intensifies the pain experience and contributes to a cycle of emotional suffering that exacerbates the burden of the disease. Baeza-Velasco et al. [7] found that patients with high levels of anxiety experience somatosensory amplification and pain catastrophizing, which increases the perception of discomfort and makes its management more difficult. This phenomenon, also observed by Schubart et al. [13], highlights the interconnection between physical and mental health in EDS and the need for approaches that include psychological support and self-care education.

The impact of pain and symptoms on the daily lives of patients with EDS is profound, affecting not only their ability to perform everyday activities but also their social and family relationships. The lack of understanding and support from the community and healthcare professionals can lead to greater social isolation and the deterioration of their personal relationships. Palomo-Toucedo et al. [14] showed that people with EDS face constant challenges due to the lack of recognition of their symptoms and the limited response from healthcare systems, which increases frustration and the negative perception of their health status. In the study of Clark et al. [2], is highlighted that the lack of knowledge about EDS among healthcare professionals contributes to fragmented care, which often delays proper diagnosis and treatment, negatively impacting patients’ perception of their health. In the qualitative study carried out by Black et al. in 2023, health care providers expressed a lack of information about their roles in EDS management. More information about symptoms related to the disease would help with early diagnosis and management [15]. De Souza-Ribeiro et al. [16] emphasized the importance of a multidisciplinary approach to improve not only medical care but also the psychological well-being and social integration of patients with EDS.

Despite the growing recognition of the severity of symptoms and the biopsychosocial impact of EDS, there is still a significant lack of understanding and effective therapeutic strategies. EDS treatment is multidisciplinary, non-specific, and mainly symptomatic. It includes pain control through medication and complementary therapies. Physiotherapy and exercise are useful to strengthen the muscles and improve the joints’ function. In addition, different orthoses are used when necessary. It should be complemented with psychological therapy, education, and lifestyle changes. Periodic monitoring by several health professionals is essential, as it affects several systems [17].

Patients often experience delays in diagnosis that postpone the start of appropriate treatments, which also contributes to a negative perception of their health status and distrust toward the healthcare system. Identifying common physical symptoms and their impact on daily life, as well as health perception, in patients with EDS, are crucial aspects for developing effective management strategies that address the complex needs of this population. Illuminating the patient experience and raising awareness of this condition will help understand how to better help patients. Addressing these challenges through a comprehensive approach can significantly improve therapeutic outcomes and the quality of life for those living with EDS. Therefore, the main objective of this study is to identify common physical symptoms, their impact on daily life, and patients’ health perceptions.

## 2. Materials and Methods

### 2.1. Study Design

The study was designed as a qualitative descriptive study based on content analysis. This methodological orientation was chosen to systematically identify and analyze patterns and themes within the data, ensuring a detailed and accurate representation of participants’ experiences and perspectives. It is not based on phenomenology but rather on a pragmatic approach to explore and describe the phenomenon under study. This study aimed at gaining an in-depth understanding of patients’ perceptions regarding the physical problems resulting from the syndrome and their impact on daily life. A qualitative approach was chosen to capture the participants’ subjective experiences and feelings, allowing for a detailed analysis of the physical, emotional, and social aspects related to their condition. The study was conducted in accordance with the COREQ (Consolidated Criteria for Reporting Qualitative Research) guidelines, which include a 32-item checklist commonly used in qualitative studies [18].

### 2.2. Participants and Context

Participants were selected through purposive sampling, recruited from various patient associations and health centers in different cities across Spain. The inclusion criteria specified that participants had to be over 18 years old, have a confirmed diagnosis of EDS, and be able to communicate effectively in Spanish. Additionally, patients with comorbidities and those with the vascular type of EDS, which could significantly interfere with the perception of the syndrome’s symptoms, were excluded. The final sample consisted of 24 patients, representing a variety of profiles in terms of age, gender, occupation, and geographic location, ensuring a broad representation of experiences.

The interviewers were a man and a woman with extensive experience in qualitative research and interviewing techniques. The entire team was composed of six researchers, two men and four women, all with more than twenty years of clinical experience in nursing and podiatric professional and academic fields.

The relationship between researchers and participants was professional and guided by ethical principles.

### 2.3. Data Collection Procedure

Individual face-to-face and in-depth interviews were conducted between April 2018 and September 2019 in four locations in Spain (A Coruña, Seville, Jaen, and Barcelona). The interviews were conducted in a quiet, private room, ensuring a comfortable and confidential environment for the participants. The average duration of the interviews was approximately 40–50 min. No other person, except a partner who asked for permission, was present during the interviews to maintain confidentiality and encourage open communication. The context of the interviews was carefully designed to minimize distractions and create a safe space for participants to share their experiences freely. A semi-structured interview guide was used, allowing the researchers to explore specific topics related to physical symptoms, the impact on daily life, and patients’ health perception, while leaving space for interviewees to openly express their experiences.

The interview guide was developed based on a thorough review of the existing literature [7,18,19,20] and adjusted after a pilot testing process. The questions focused on key aspects outlined in Table 1.

All interviews were audio recorded with the prior consent of the participants and then fully transcribed. The transcripts were reviewed to ensure accuracy and compared with field notes taken during the interviews, allowing for greater fidelity in the interpretation of the data. Interobserver variability was minimized by ensuring that both interviewers received prior training to conduct the interviews properly. Importantly, neither interviewer had any professional or personal relationship with the patients. The interviews averaged 41 min in duration (ranging from 27 to 55 min). For inclusion in this paper, the interviews were translated into English by a native speaker, and then retranslated into Spanish to ensure translation accuracy. Any discrepancies were resolved by reviewing the original audio recordings. The final themes were agreed upon by all researchers through consensus.

### 2.4. Data Analysis

The data analysis was conducted using a thematic analysis approach, following Braun and Clarke’s method [21]. This method was chosen for its effectiveness in identifying patterns and themes within qualitative data, allowing for a rich and in-depth interpretation of patients’ experiences. The data were analyzed by F.L.-L., an experienced nurse in quality research who was not involved in conducting the interviews. The analysis followed a systematic approach to ensure reliability and consistency.

The analysis process was carried out in several stages:

#### 2.4.1. Familiarization with the Data

At this stage, an intensive reading of all interview transcripts was undertaken. This process allowed us to gain an in-depth understanding of the data and begin to identify possible emerging patterns. During the reading, initial notes were taken on recurrent aspects, such as the most frequently mentioned physical symptoms and impacts on daily life, and patients’ perception of health.

#### 2.4.2. Initial Code Generation

Interesting features of the data were systematically coded across all transcripts. Codes were assigned to relevant segments of text representing key aspects such as ‘chronic pain’, ‘fatigue’, ‘limitations in daily activities’, ‘impact on social life’, ‘perception of health’, and other relevant factors. This phase involved both manual coding and the use of qualitative software, NVivo 1.0, to analyze data (QSR International, Melbourne, Australia) to ensure complete and accurate coverage of the data. This software allowed large volumes of data to be handled in an efficient and structured manner, which facilitated the identification of patterns and the generation of themes.

#### 2.4.3. Searching for Themes

After coding the data, the codes were grouped together to form broader themes. This process involved organizing the codes into preliminary themes that reflected central aspects of patients’ experiences.

#### 2.4.4. Review of Themes

In this phase, the preliminary themes were reviewed to ensure that they formed a consistent and meaningful pattern in the data. This process included comparing the themes with the original data to verify that the identified themes accurately captured the experiences expressed by the participants. Themes were removed, combined, or split as necessary to improve clarity and consistency.

- For example, the themes ‘chronic pain’ and ‘fatigue’ were combined into a broader theme of ‘Predominant physical symptoms’ that included sub-themes for each specific symptom.
- The theme ‘Impact on social and family relationships’ was refined to include sub-themes such as ‘Social isolation’ and ‘Family support’.

#### 2.4.5. Topic Definition and Naming

Each theme was clearly defined and assigned a name that accurately reflected its content and scope. The final themes were described as follows:

- Common physical symptoms
- Impact on daily life
- Impact on social and family relationships
- Perception of health and emotional well-being

Reflexivity was consistently upheld throughout the study by managing pre-existing assumptions and beliefs via reflective journaling. Themes were driven by the data, with minimal interpretive bias. Rigor and trustworthiness were ensured through a meticulous analysis process. The identified themes were reviewed by the authors, resonated with patients, and aligned with their lived experiences.

### 2.5. Ethical Considerations

This study was authorized by the Research Ethical Committee of Virgen Macarena and Virgen del Rocío Hospitals with number 1754-N-17 and obtained a favorable evaluation, ensuring compliance with all applicable ethical and legal regulations. Informed consent was obtained from all participants prior to data collection, ensuring that they fully understood the purpose of the study, the use of the data, and their rights as participants.

Data confidentiality was always maintained. Personal data were anonymized, and each participant was given a unique code to ensure their privacy. Audio recordings and transcripts were stored on a secure server, accessible only by the research team, and will be destroyed after a defined period of time, in accordance with legal guidelines. This research was funded by “VI PLAN PROPIO DE INVESTIGACIÓN Y TRANSFERENCIA—US 2018” of the University of Seville.

## 3. Results

Thirty-one individuals were invited to participate in this study. However, five patients declined participation, primarily due to scheduling conflicts that prevented them from being available for the interview. Two of them had vascular EDS type, so they were not included in the study, and the rest had diagnoses of classic or hypermobile EDS. The data presented were gathered from 24 individual interviews, achieving information saturation. The sociodemographic characteristics of the participants are outlined in Table 2.

The final report was developed by synthesizing the identified themes and sub-themes (Table 3) and supporting them with verbatims from the interviews, which illustrate and bring to life the themes described. The verbatims were carefully selected to ensure that they effectively represented the experiences and feelings expressed by the participants in relation to each theme.

### 3.1. Common Physical Symptoms

This theme includes patients’ experience of chronic pain, extreme fatigue, and joint problems, which are the most-commonly reported symptoms that significantly affect their daily lives. The physical symptoms most frequently mentioned by patients in interviews include chronic pain, extreme fatigue, joint problems, and sleep-related problems.

#### 3.1.1. Chronic Pain and Fatigue

Chronic pain and fatigue are recurring symptoms mentioned by almost all participants. These symptoms significantly impact their ability to carry out daily activities, affecting various joints and causing debilitating fatigue. These are the most debilitating symptoms that limit their ability to perform simple tasks and participate in social activities.

*“Fatigue and pain are the most.”*

(P 6)

*“Pain I of course for me having pain was like normal.”*

(P 17)

*“Especially the pain because it is quite disabling every day.”*

(P 23)

#### 3.1.2. Joint Problems

Patients often mention joint problems, including stiffness, recurrent subluxations, and difficulty moving especially in the mornings, due to muscle stiffness.

*“I have a lot of stiffness, and I take time...to get my body going.”*

(P 6)

*“Especially in the joints let’s say, in the ankles, knees.”*

(P 17)

*“The knees... since I can’t stay in the same posture for a long time, I move them all the time.”*

(P 21)

#### 3.1.3. Digestive Problems

Digestive problems are also mentioned as an additional symptom, present in some patients, that affect their overall well-being. Although not all patients mention them, some do.

*“I’m having tests and so on I’ve had a long time with.... Since I was little with stomach pains.”*

(P 10)

#### 3.1.4. Vascular Problems

Some patients mention problems related to circulation, such as Raynaud’s syndrome, which causes them additional pain and complications.

*“Raynaud’s syndrome, which also because our hands are often swollen, they become bluish, pain, varicose veins.”*

(P 7)

#### 3.1.5. Sleep Problems

Sleep disturbances, often caused by pain, are another co-occurring symptom, compounding tiredness and fatigue.

*“Pain makes you wake up a lot...then I end up waking up with the pain.”*

(P 23)

### 3.2. Impact on Daily Life

This theme captures how physical symptoms limit patients’ ability to perform daily activities, work, and maintain a regular routine. The above symptoms profoundly affect patients’ ability to lead a normal daily life, limiting their activities and affecting their social and family relationships. Everyday activities such as dressing, working, or even walking become significant challenges.

#### Difficulties in Carrying Out Daily Activities

This theme covers how the disease affects social and family interactions, including social isolation and misunderstanding by others. Pain and fatigue limit patients’ ability to perform basic daily tasks, such as working, studying, or maintaining a regular routine.

*“It’s like you climb a mountain but every day.”*

(P 17)

*“It affects me in everything I can imagine about my life.”*

(P 6)

*“Driving that is one of the things that I deal with the worst and it’s one of the things that can create a little bit more problems with my partner.”*

(P 7)

### 3.3. Impact on Social and Family Relationships

This theme covers how the disease affects social and family interactions, including social isolation and misunderstanding by others. Social and family relationships are affected due to the patients’ physical limitations. Some mention incomprehension on the part of their environment, which increases their isolation as they are unable to fulfill social or family commitments due to their physical condition.

*“My social life has diminished tremendously. If I can stay days and days at a time in my house locked up, that’s where I am better off.”*

(P 21)

*“You lose contact...people end up leaving me and socially this.... well, there are some people who are, are there and with them I must count on, but others no longer.”*

(P 6)

*“For example, with my daily life... I just get up to go to practice because I don’t get up... I can’t get up earlier to do my things.”*

(P 10)

### 3.4. Health Perception and Well-Being

This theme explores how patients perceive their overall health, including the stress, uncertainty and emotional challenges associated with living with the syndrome. The perception of health among patients is marked by a high degree of uncertainty, stress, and frustration, due to the unpredictable and chronic nature of their symptoms that has a broad impact on their lives.

#### 3.4.1. Uncertainty and Stress

Patients express great concern about their future and how symptoms will continue to affect their lives. This uncertainty increases stress levels.

*“What affects me the most about this disease first is the tiredness...and the second thing is the emotional fear that they transmit to you through this disease.”*

(P 10)

*“The stress I notice that it accumulates... and I have a job that is also intense, and it does get to me to block me.”*

(P 17)

*“Psychologically... I have spent periods crying and not wanting to see anyone.”*

(P 21)

*“I never know if tomorrow I’m going to get up, I never know.”*

(P 6)

#### 3.4.2. Frustration and Hopelessness

Some patients express feelings of hopelessness due to the lack of complete relief of symptoms, even with medication.

*“There are times when you take some … Like … The strongest thing, right? Maybe they send you an opiate, and you see that it doesn’t do it for you.”*

(P 7)

## 4. Discussion

The aim of this research was to investigate the most common symptoms associated with EDS. The aim was also to find out the perceived impact on daily life, social relations, and the work environment of those affected. The information obtained reveals that these symptoms generate a great physical limitation in the performance of daily life, being mainly chronic pain, fatigue, joint disorders, digestive problems, vascular problems, and sleep disturbance. These physical afflictions, in turn, are combined with mental health concerns significantly affecting their quality of life.

However, pain, whether acute or chronic, is the symptom most frequently referred to in all the articles as the most prevalent and most limiting, with varying severity and in different joints. Guerrieri et al. [22] reported that 80% of patients with EDS over 40 years of age have pain, which can be of various types: nociceptive, partly associated with hypermobility; and neuropathic, related to loss of proprioception, both of which present later. This reinforces the idea that pain in EDS patients is not only due to joint hypermobility but also includes dysfunction of the nerve fibers responsible for processing thermal and nociceptive sensations. This may explain why some patients experience severe and persistent nociplastic pain, even in the absence of obvious joint damage [23].

The worldwide prevalence of EDS is 1 to 9 affected per 100,000 for the classic type and 1 to 5 per 10,000 for the hypermobility type. Therefore, according to the European Union Regulation on Orphan Medicinal Products (1999), EDS is considered a rare disease because it affects no more than 1 person per 2000 in the European population [24].

However, following the 2017 revision of the EDS classification system [25], there is a tendency to study classic and hypermobile EDS syndrome together with hypermobility spectrum disorders, differentiating them from other EDS types [4]. Thus, it is possible to extract clinical feature similarities in both conditions [10,11] from the perspective of the diagnostic delay suffered by these patients [2,16,26]. In general, common therapeutic action protocols were proposed [27,28]. Schubart et al. [29] concluded that both disorders imply a very high and additional cost in health that is not paid for by the public health services. From these points of view, and since there is a consensus of underdiagnosis, we could say that the combined prevalence is high and that it should not be considered a ‘rare disease’ [2,11].

This statement is supported by recent studies such as Demmler et al. [11], which report a global prevalence of hEDS/HSD of 194/100,000 in Wales, or Clark et al. [2], who affirm that joint hypermobility affects around 30% of the population of the United Kingdom.

In our study, only patients with a confirmed diagnosis of the disease participated, discarding individuals with clinical suspicion or under study period.

Most authors establish the relationship between biomechanical dysfunctions associated with hyperlaxity and hypermobility and chronic pain [16]. The results found by Murray et al. [3] revealed that 99% of respondents with hEDS had joint pain in some part of the body. In our study, patients with musculoskeletal involvement also expressed joint problems such as stiffness, mainly morning stiffness characteristic of rheumatic processes. This finding was also detected by Viswanath et al. [30].

As an emerging idea, there is the relationship between pain, hyperlaxity, and mastocytosis (Mast Cell Activation Syndrome, MCAS), which requires further scientific evidence [11,29,31,32].

Chronic fatigue was widely described along with pain [2,5,13]. Murray et al. [3] reported that 82% of his sample claimed to suffer from it, while Teran-Wodzinski and Kumar [4] related that the prevalence of both was 98%. In general, it highlights the great implication in the reduction of quality of life, as it reduces the ability to perform simple tasks of daily life, as it is related to physical disability. Thus, Anderson and Lane [10], in a systematic review, found that fatigue can be more debilitating than pain. This is consistent with the experiences reported by our participants in the interviews.

Sleep disturbance and fatigue are common in patients with EDS [3,33]. Combined with pain and fatigue, it impacts on physical well-being [13] and at the same time has a significant negative emotional impact, as seen in the high prevalence of anxiety disorders and depression among patients [2]. Our findings confirm this relationship between pain and sleep disorders, as well as the impact on physical well-being. Lamari et al. [34], established that nocturnal insomnia and unrefreshing sleep is common in patients with hypermobility.

There is evidence that the quality of life of affected people scores worse than that of healthy individuals [2,6,12,35]. Thus, Orenius et al. [33] reported that people with hypermobile EDS showed significantly lower total scores than the control group. The same author concluded that this negatively affects the overall functional ability of these individuals. In several qualitative studies, patients interviewed endorsed this evidence from the perspective of their life experience, confirming the impact of physical symptoms on their day-to-day lives.

Moreover, these debilitating symptoms worsen over time [13,32,36], partly because there is no effective treatment with evidence of helping reduce symptoms overall.

In our study, the patients interviewed expressed the physical limitations they have in carrying out daily activities, whether in the domestic sphere, studying, or working. The simple act of driving is described as stressful for one of our respondents, making their mobility difficult. This finding is supported by the results of Murray et al. [3], who, after surveying 466 patients, found that 53% of hEDS patients reported that their disorder affected their performance at work and study.

Schubart et al. [13] and Murray et al. [3] stated that patients generally continue to work despite the disability or limitation, adapting the characteristics of the work environment.

Chunchin et al. [32] concluded that fear of falling is prevalent and may play an important role in the establishment and worsening of disability in people with hypermobility, as well as problems with joint instability, proprioception, and impaired gait and balance. Our patients often report joint problems that lead to difficulty in moving, as stated by these authors.

Social and family relationships are severely restricted by the physical limitations of those affected [5], as our participants report in their life experiences. In addition, some mention social incomprehension, partly because the most disabling symptoms are not objectifiable, such as pain or fatigue. This is exacerbated by the lack of a clear diagnosis and conclusive clinical evidence. De Baets et al. [26] concluded that persons with hEDS have a significantly lower rate of social participation than healthy individuals.

All the mentioned aspects have an impact on the perception of health and well-being. In our study, patients expressed uncertainty about their future, either at work or related to the evolution of the disease itself, which in turn exacerbates stress and emotional disturbances. Giroux et al. [37] found the same findings in high school students with EDS.

We believe that the dissemination of the results of this study will help clinicians and researchers to better understand affected people, which will have a positive impact on patients, since a better understanding of this syndrome will help in earlier diagnosis and effective management of the EDS.

A limitation of this qualitative study is the lack of diversity in the perspectives captured, as only patients with the classical and hypermobile EDS subtypes were included, which may limit the full understanding of the experiences of other subtypes of the syndrome, such as vascular, which present different clinical features. Future studies should include a more diverse sample to better capture the heterogeneity of EDS and ensure representativeness of findings across the affected population.

Another limitation of this study is the lack of data triangulation, as it relied solely on individual semi-structured interviews. The inclusion of other sources of information, such as medical records, patient diaries or questionnaires, would have allowed us to contrast and corroborate the findings, improving the validity of the study. Future studies should consider triangulation to obtain a more complete picture and reduce possible biases arising from participants’ self-perceptions.

## 5. Conclusions

In conclusion, patients diagnosed with non-vascular EDS suffer chronic symptoms such as pain, fatigue, and physical limitations that hinder their daily activities and interpersonal relationships, causing a profound impact on their quality of life. In addition, uncertainty about the future and frustration about the lack of complete relief aggravate their emotional well-being, contributing to a state of stress and discomfort. It is crucial to increase the visibility of this syndrome and, furthermore, to address their needs, not only from a multidisciplinary approach, but also by providing emotional and social support to improve their perceived quality of life.

## Figures and Tables

**Table 1:** Interview guide.

What physical symptoms do you associate with your illness, and how do these affect your overall health? How do these symptoms impact your daily life, including your everyday activities and social relationships? What is your overall perception of your health since developing this condition?

**Table 2:** Participants’ sociodemographic characteristics relating to participants of interviews.

Participant	Age	Gender	Education	**Employment**
P1	42	Male	Primary	Autonomous, assembler
P2	46	Female	University	Engineer
P3	38	Female	Primary	Elder caregiver
P4	18	Male	Secondary	Student
P5	49	Female	Secondary	Administrative
P6	47	Female	University	Executive
P7	37	Female	Primary	Hairdresser
P8	48	Female	Secondary	Farmer
P9	53	Female	Secondary	Administrative
P10	25	Male	Secondary	Student
P11	54	Female	Secondary	Occupational therapist
P12	41	Female	Secondary	Administrative
P13	42	Male	University	Pharmacist
P14	31	Female	University	Administrative
P15	57	Female	Secondary	Functionary
P16	56	Female	Primary	Fishmonger
P17	46	Female	University	Biologist
P18	33	Female	Secondary	Marketing
P19	48	Female	University	Administrative
P20	40	Female	University	Teacher
P21	45	Female	University	Bank clerk
P22	24	Male	Secondary	Student
P23	20	Female	Secondary	Student
P24	57	Female	Secondary	Nursing Assistant

**Table 3:** Themes and subtheme.

1. Common physical symptoms
1.1. Chronic pain and fatigue 1.2. Joint problems 1.3. Digestive problems 1.4. Vascular problems 1.5. Sleep problems
**2. Impact on daily life**
2.1. Difficulties in carrying out daily activities
**3. Impact on social and family relationships**
**4. Health perception and well-being**
4.1. Uncertainty and Stress 4.2. Frustration and hopelessness

## Data Availability

The data presented in this study are not available due to privacy.
